# Correction: Repair of multiple simultaneous double-strand breaks causes bursts of genome-wide clustered hypermutation

**DOI:** 10.1371/journal.pbio.3000695

**Published:** 2020-03-06

**Authors:** Cynthia J. Sakofsky, Natalie Saini, Leszek J. Klimczak, Kin Chan, Ewa P. Malc, Piotr A. Mieczkowski, Adam B. Burkholder, David Fargo, Dmitry A. Gordenin

In Fig 4 the diploid and haploid bars are swapped. The x-axis labels are correct but the bar previously displayed for ‘diploid’ should appear above ‘haploid’ and vice versa. The authors have provided a corrected Fig 4 here.

**Fig 4:**
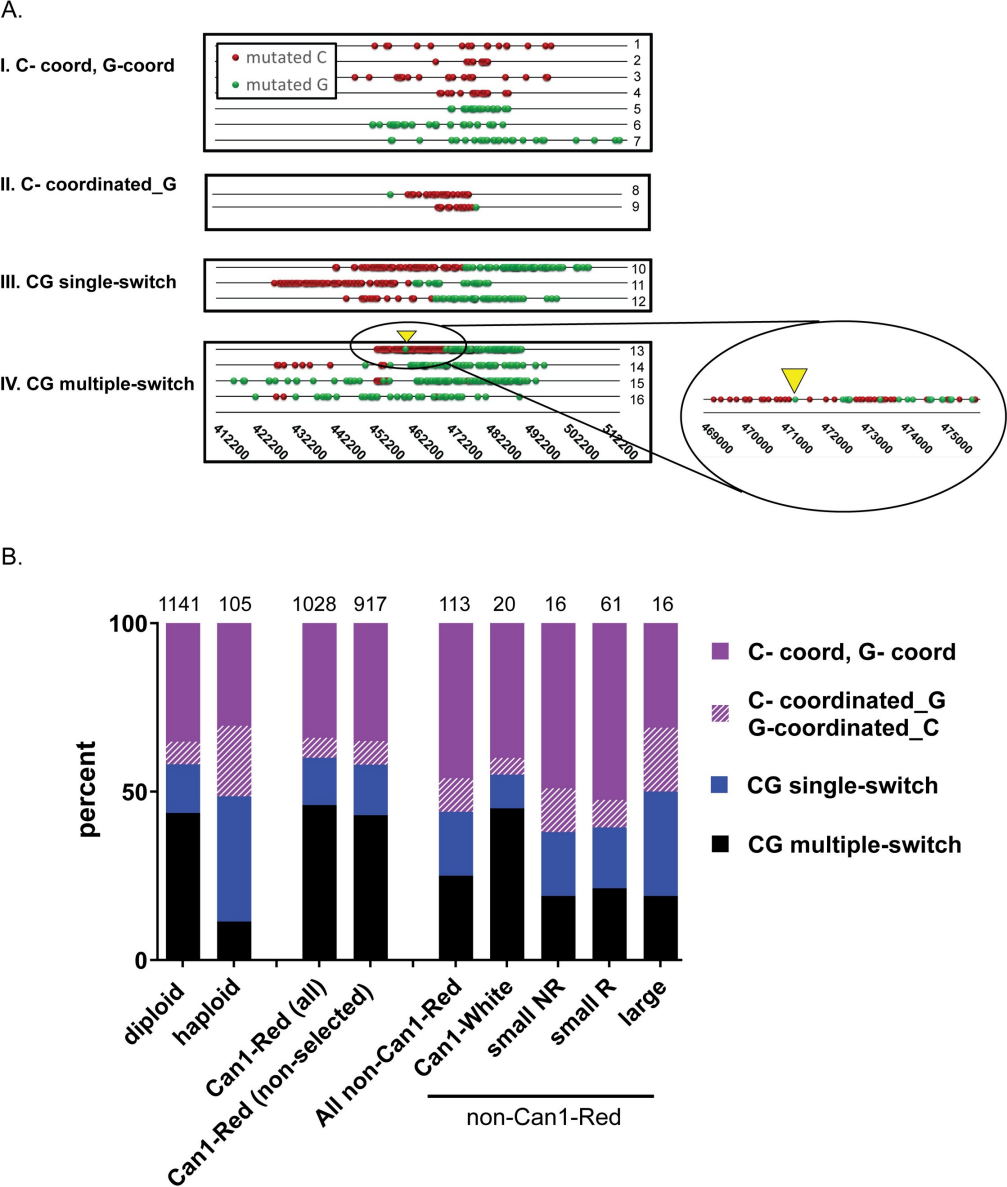
Cluster types in gamma-exposed yeast. (A) Example of selected clusters overlapping with the triple reporter on chromosome 2 found in WT Can1-Red diploid or haploid gamma-exposed yeast. Symbols include: “red spheres” = mutated C’s, “green spheres” = mutated G’s. Each numbered line (1–16) shows a unique cluster. Information about these clusters and their derivative strains can be found in S2G Table and S1 Data. Four cluster types are illustrated: (I) C-coordinated or G-coordinated clusters, comprised exclusively of either mutated C’s or mutated G’s on the top strand; (II) C-coordinated clusters adjacent to a single G. The terminal G in these clusters may have originated from the same mutagenic event that formed the C-coordinated portion of the cluster or it could be an unlinked random, scattered mutation. Since we could not distinguish between these two possibilities, they are represented here as a separate class. G-coordinated clusters adjacent to a single C were also detected (not illustrated); (III) CG single-switch clusters include clusters that have a single switch in coordination of C to G in the top strand in a 5′ to 3′ direction (called 5′ C to G single-switch) or have a switch from G to C in a 5′ to 3′ direction (called 5′ G to C single-switch) (not illustrated); (IV) CG multiple-switch clusters having alternating mutated C’s and G’s in the top strand. A portion of cluster number 13 is magnified to illustrate the finer detail of CG switching. Yellow triangle in magnified portion shows corresponding position in unmagnified view. A complete list of cluster categories reflecting strand assignment of mutations is provided in Materials and Methods. (B) The distribution of selected and nonselected gamma-induced cluster types formed from >3 C and/or G mutations in a cluster identified in the following colony types: (i) diploid = selected and nonselected clusters from all WT diploid isolates (Can1-Red and non-Can1-Red isolates); (ii) haploid = selected and nonselected clusters from Can1-Red WT haploid isolates; (iii) Can1-Red all = selected and nonselected clusters from Can1-Red isolates; (iv) Can1-Red (nonselected) = only nonselected clusters from Can1-Red isolates; and (v) non-Can1-Red = clusters from all non-Can1-Red isolates including Can1-White, small NR (not rearranged), small R (rearranged), and large colonies. The four classes of clusters shown are detailed in (A) and include (i) C-coord, G-coord: C- or G-coordinated clusters; (ii) C-coordinated_G, G-coordinated_C: C-coordinated clusters adjacent to a single G or G-coordinated clusters adjacent to a single C, respectively; (iii) CG single-switch; and (iv) CG multiple-switch. Source data in S2H Table. (See also S1, S2 and S3 Tables, S3 Fig). CG, C- and/or G-containing; WT, wild type.
