# Supplementary figures and images for: Repair of multiple simultaneous double-strand breaks causes bursts of genome-wide clustered hypermutation
Source: PLoS Biol. 2019 Sep 30;17(9):e3000464. doi: 10.1371/journal.pbio.3000464 (PMC6786661; doi:10.1371/journal.pbio.3000464)

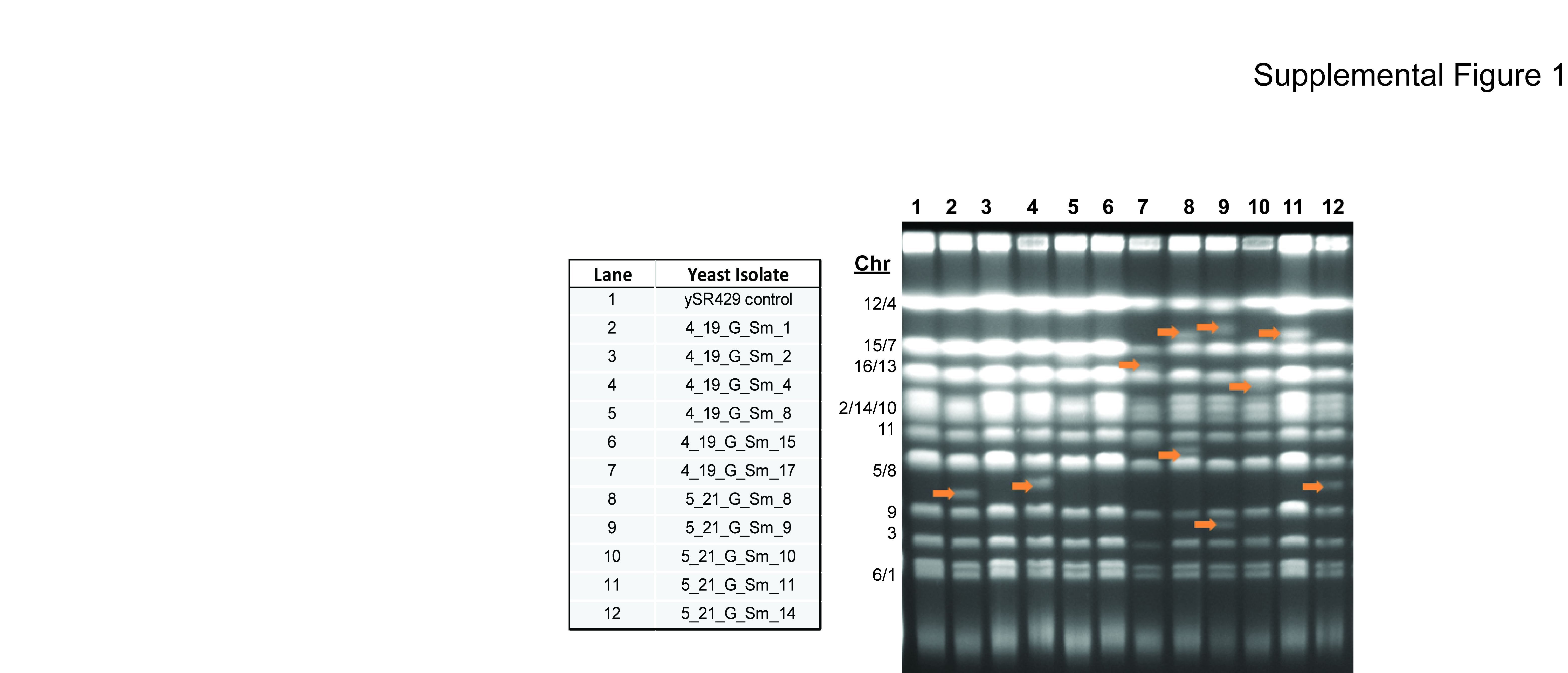

Supplement: S1 Fig — Examples of small WT diploid colonies with chromosomal rearrangements detected by PFGE. Lane 1 shows control strain with no rearrangements. Lanes 2, 4, and 7–12 show isolates with rearranged chromosomes as indicated by orange arrows. Rearranged chromosomes have positions that deviate from those in the control strain. Chromosome 1–16 positions are indicated adjacent to gel image. PFGE, pulse-field gel electrophoresis; WT, wild type. (TIF) [file pbio.3000464.s001.tif]

A.

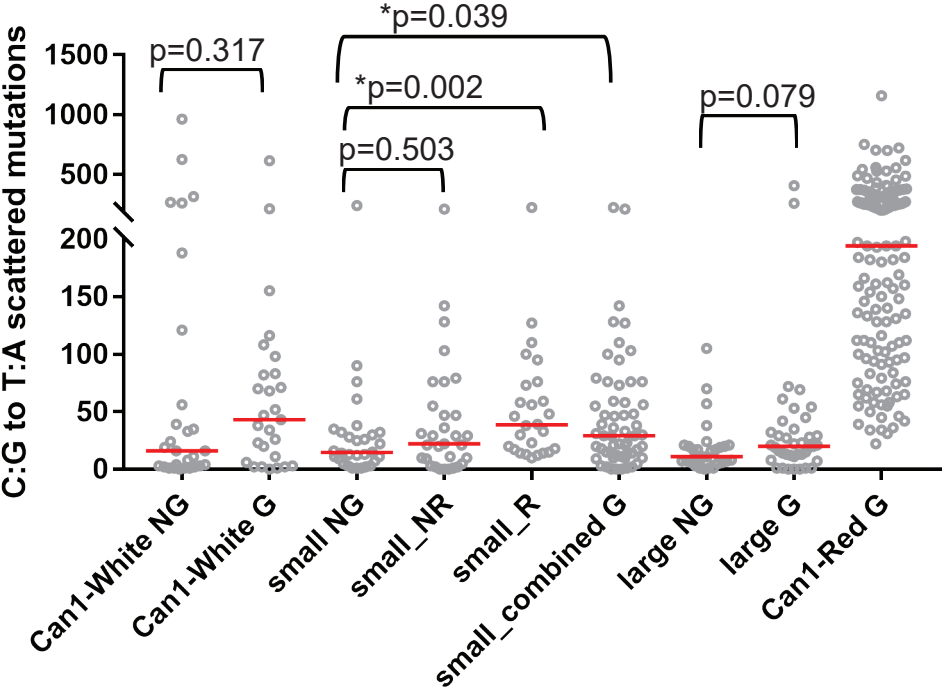

B.

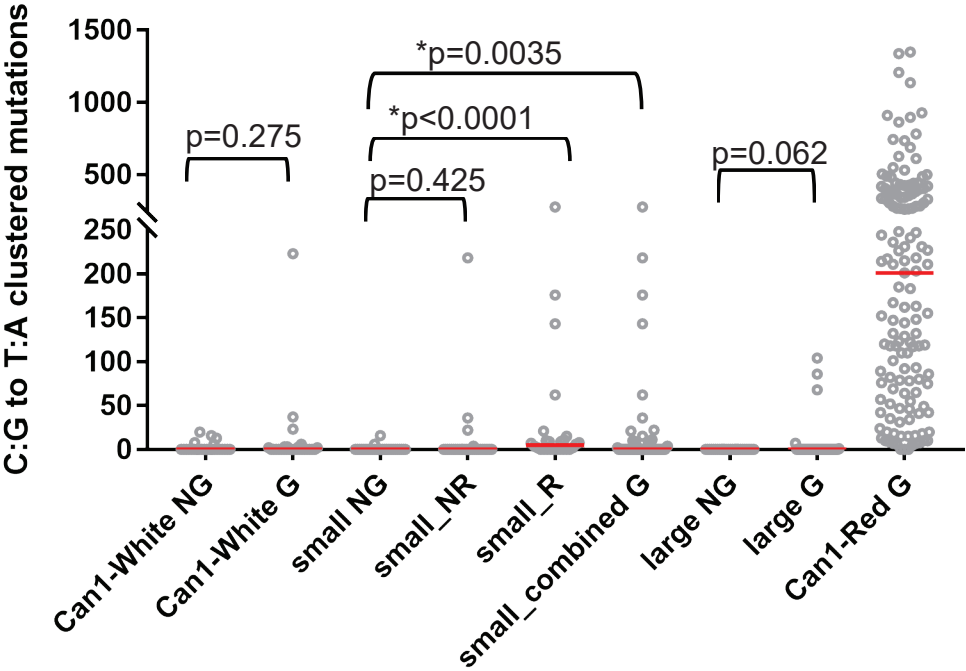

Supplement: S2 Fig — (A) The total numbers of scattered C:G to T:A mutations per isolate in WT diploid yeast. Source data in S1F Table. (B) The total numbers of clustered C:G to T:A mutations (from selected and nonselected clusters) per isolate in WT diploid yeast. Source data in S1F Table. Gray circles indicate an individual isolate, and red lines show median values. NG, no gamma exposure; G, gamma exposure (80 krad); NR, isolates with no rearranged chromosomes; small, small colonies; small_combined, small colonies with and without rearrangements combined; large, large colonies. P-values were calculated from Mann–Whitney t test shown above groups. “*” indicates significant P-values (<0.05). APOBEC, apolipoprotein B mRNA editing enzyme, catalytic polypeptide-like; WT, wild type. (PDF) [file pbio.3000464.s002.pdf]

Supplemental Figure 3 (A, B, C)

Haploid

Diploid

Cancer

A.

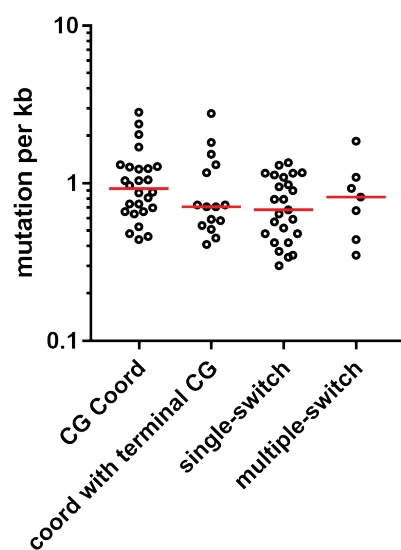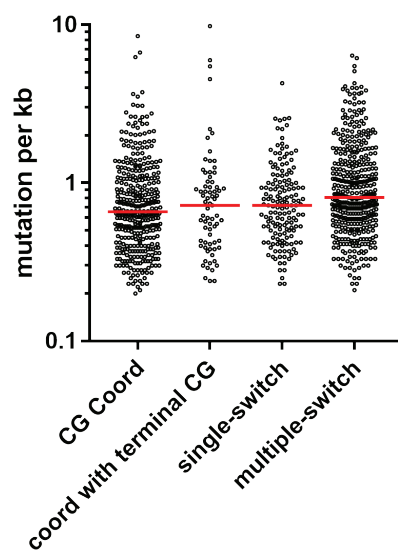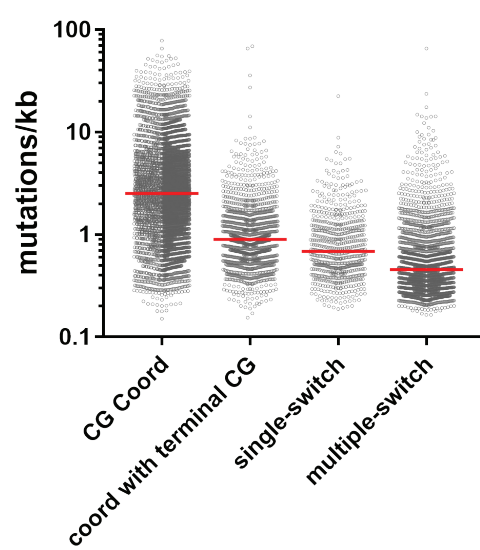

B.

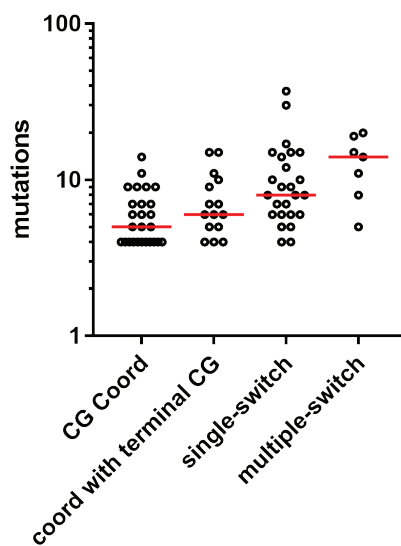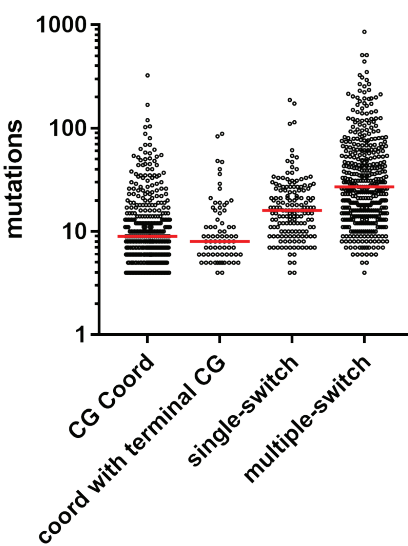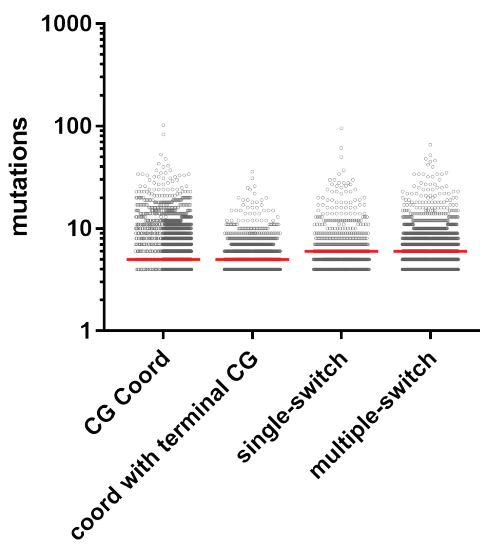

C.

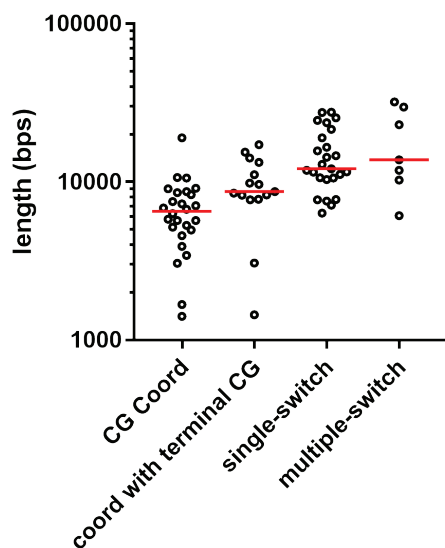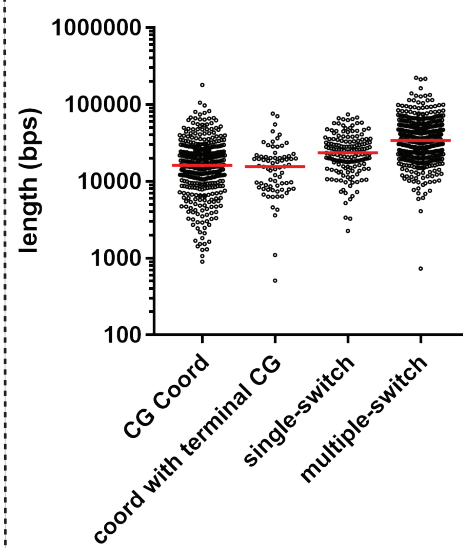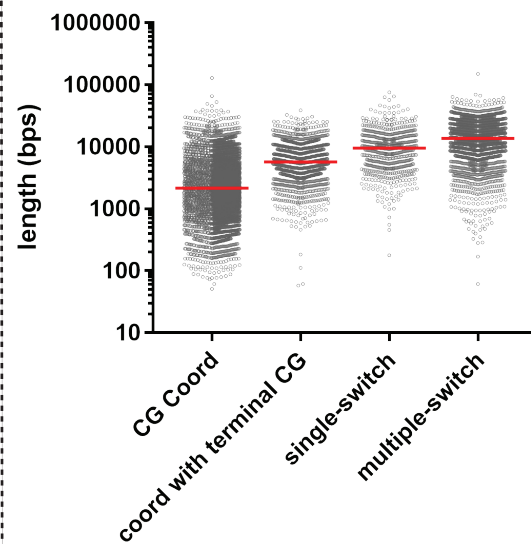

Supplemental Figure 3 (D)

D.

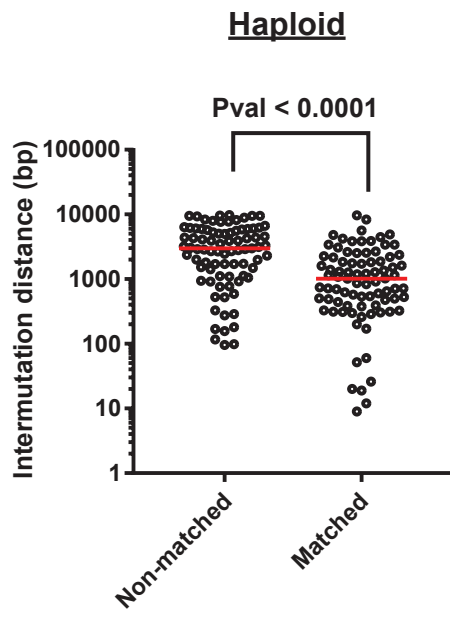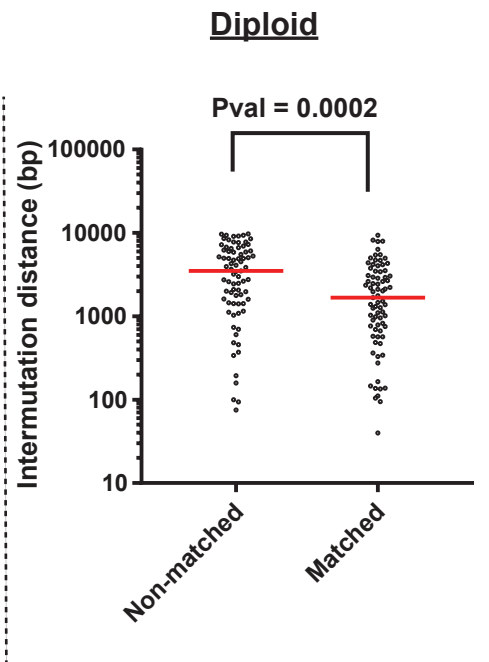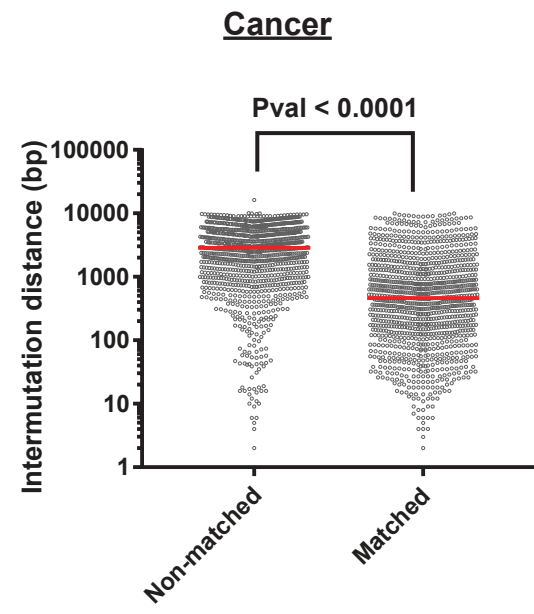

Supplement: S3 Fig — For all graphs in this figure, red lines show median values and black circles show individual clusters in haploid yeast and in diploid yeast (WT strains, nonselected clusters in panels A, B, and C and all clusters in all genotypes in panel D) or clusters in tumor samples. Only CG clusters with >3 mutations were used to make graphs. Clusters are divided into four main types: (i) CG coord, C- or G-coordinated; (ii) coord with terminal CG, C-coordinated clusters adjacent to a single G or G-coordinated clusters adjacent to a single C; (iii) single-switch clusters; and (iv) multiple-switch clusters. Details about cluster types are in Fig 4. (A) Mutation density of clustered mutations. Source data are in “Mutation_Density_per_kb” column of S3A Table for yeast and in “Mutation_Density_per_kb” column of S5A Table for cancer (see S3 and S5 Tables descriptions for details). (B) Size of clusters, i.e., the number of mutations in the cluster. Source data are in “Cluster_Size_Complexes” column of S3A Table for yeast and of S5A Table for cancer. (See S3 and S5 Tables descriptions for details.) (C) Cluster length from the position of the first nucleotide to the last nucleotide in the mutation cluster; reported in bps. Source data are in “Cluster_Length” column of S3A Table for yeast and of S5A Table for cancer. (See S3 and S5 Tables descriptions for details). (D) Distance between the terminal matched or nonmatched residues and the preceding mutated residue in each cluster designated as “coord with terminal CG.” “Matched” and “nonmatched” correspond to the columns “Distance_terminal_non_matched” and “Distance_terminal_matched” in the S3 Table and S5 Table for yeasts and cancers, respectively. P-values depicted over the graphs were calculated using a two-tailed Mann–Whitney test. Source data in S3B Table and in S5B Table. CG, C- and/or G-containing; WT, wild type. (PDF) [file pbio.3000464.s003.pdf]

A.

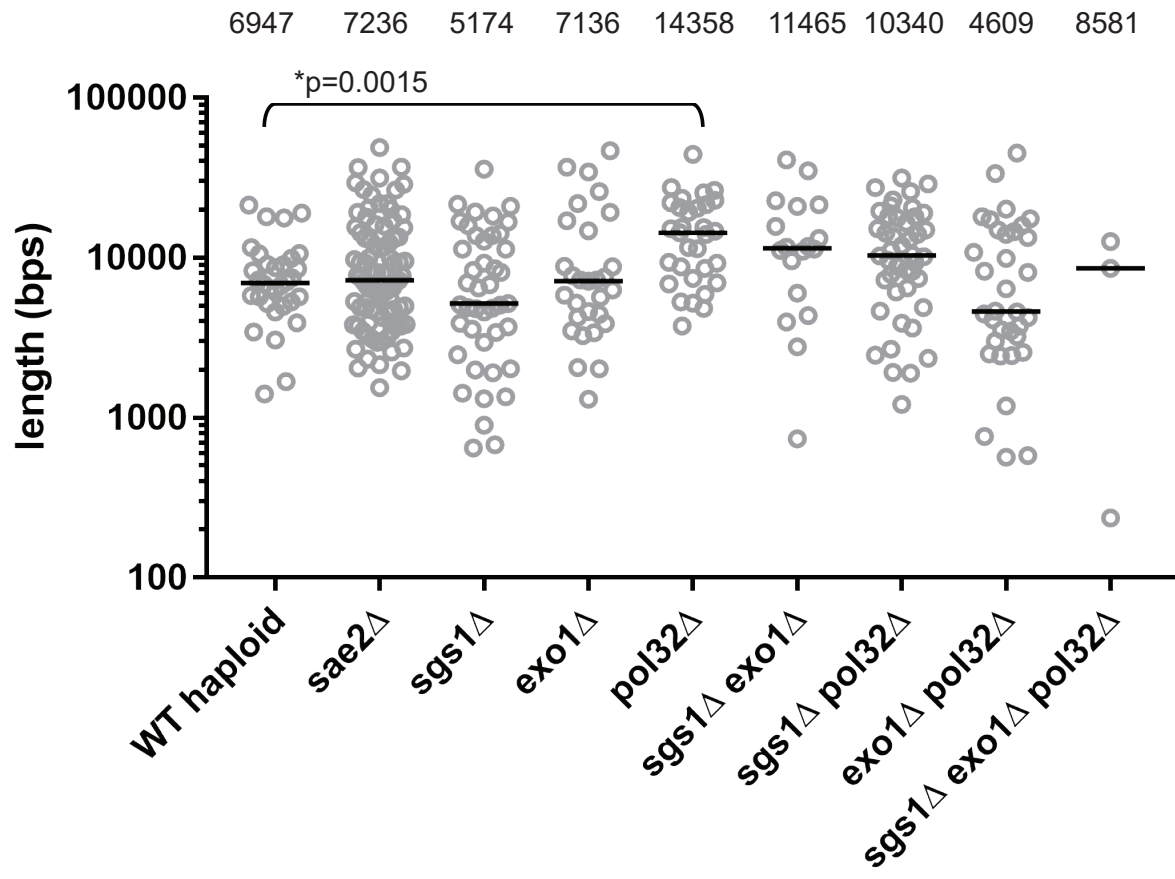

B.

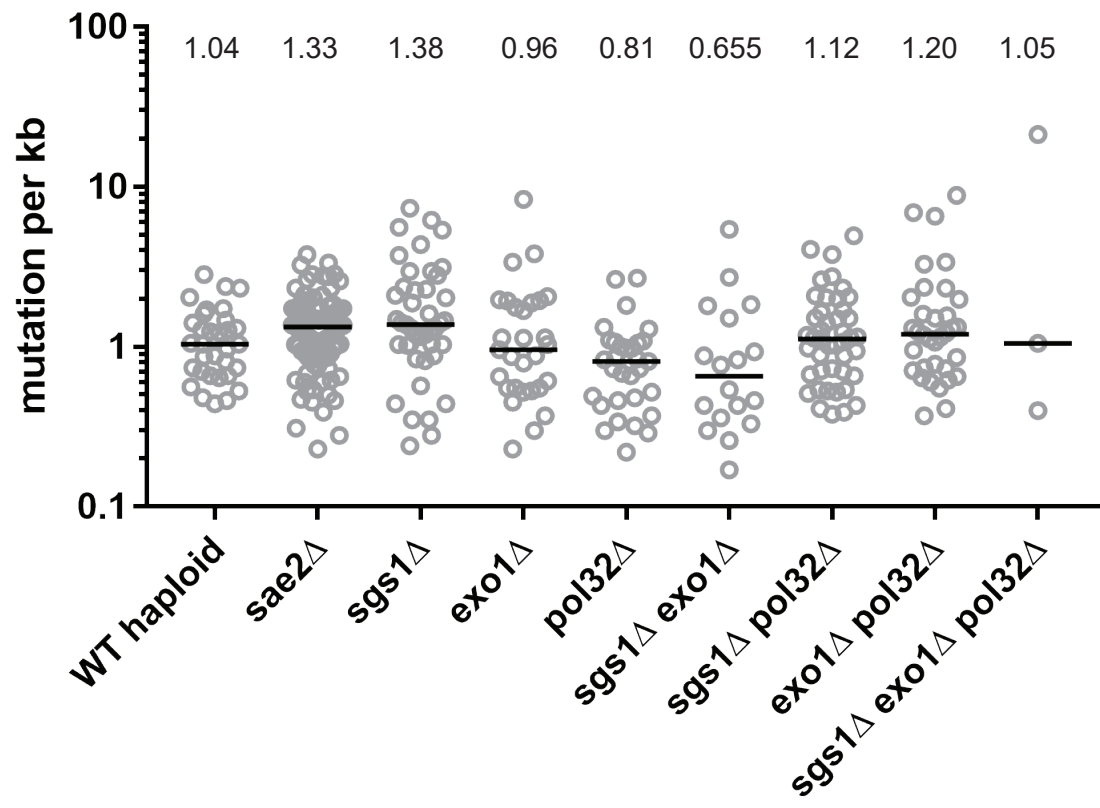

Supplement: S4 Fig — (A) The distribution of cluster lengths for C- or G-coordinated with clusters >3 mutations in haploid strains. Source data in S2J Table. (B) The distribution of mutation density of clusters C- or G-coordinated with clusters >3 mutations in haploid strains. For all graphs, gray circles indicate individual clusters, and black lines show median values. Median values are written above each distribution. Mann–Whitney two-tailed t test with Bonferroni correction for multiple hypothesis testing showed a significant increase in cluster lengths in pol32 mutants as compared to WT, and P-value is shown on graph (see S2J Table for more details). There were no significant differences between clusters in sgs1Δ exo1Δ from 20 krad versus 40 krad irradiation, and thus, clusters in this strain were pooled together. Source data in S2J Table. WT, wild type. (PDF) [file pbio.3000464.s004.pdf]

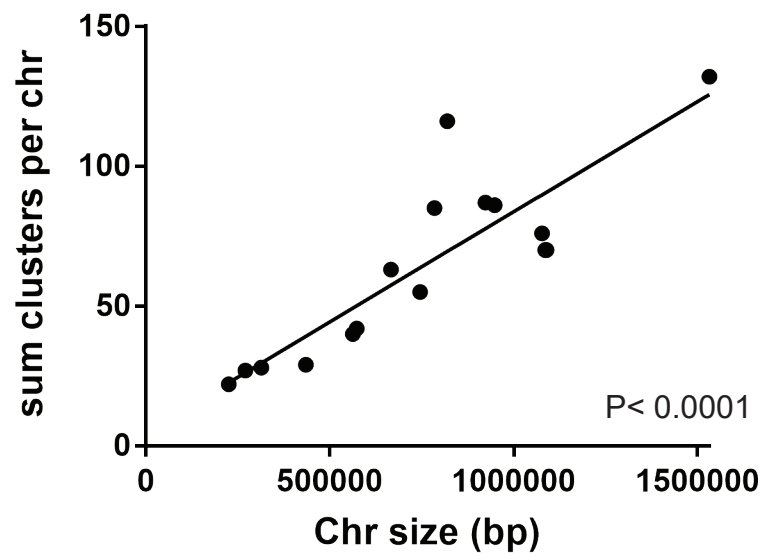

Supplement: S6 Fig — The distribution of nonselected clusters across all 16 yeast chromosomes in WT diploid yeast. P-values from linear regression analysis shows that clusters are spread across yeast chromosomes proportionately to the size of the chromosomes (P < 0.0001). Source data for this figure are shown in S4F Table. WT, wild type. (PDF) [file pbio.3000464.s006.pdf]
