# Supplementary material for: Repair of multiple simultaneous double-strand breaks causes bursts of genome-wide clustered hypermutation
Source: PLoS Biol. 2019 Sep 30;17(9):e3000464. doi: 10.1371/journal.pbio.3000464 (PMC6786661; doi:10.1371/journal.pbio.3000464)
Supplement: S5 Fig — (A) Coverage maps of sequencing reads in sgs1Δ exo1Δ yeast isolate (164_sgsexo_28) showing a copy number increase from coordinates 524,672 to 601,156 in chromosome 14, with an overlapping cluster shown as a red horizontal line above the graph. Cluster details are shown below the graph. (B) Coverage maps of sequencing reads in sgs1Δ exo1Δ yeast isolate (165_sgsexo_4) showing a copy number increase from coordinates 1,328 to 136,201 in chromosome 8 with an overlapping cluster shown as a red horizontal line above the graph. Cluster details are shown below the graph. (C) Coverage maps of sequencing reads in sgs1Δ pol32Δ yeast isolate (227_sgs1pol32_8) showing a copy number increase from coordinates 6,208 to 136,761 in chromosome 5 with an overlapping cluster shown as a red horizontal line above the graph. Cluster details are shown below the graph. Complete list of all CNVs detected in haploid isolates as well as of mutation clusters overlapping with CNVs is in S4E Table. CNV, copy number variation. (PDF) [file pbio.3000464.s005.pdf]

Supplemental Figure 5

A.

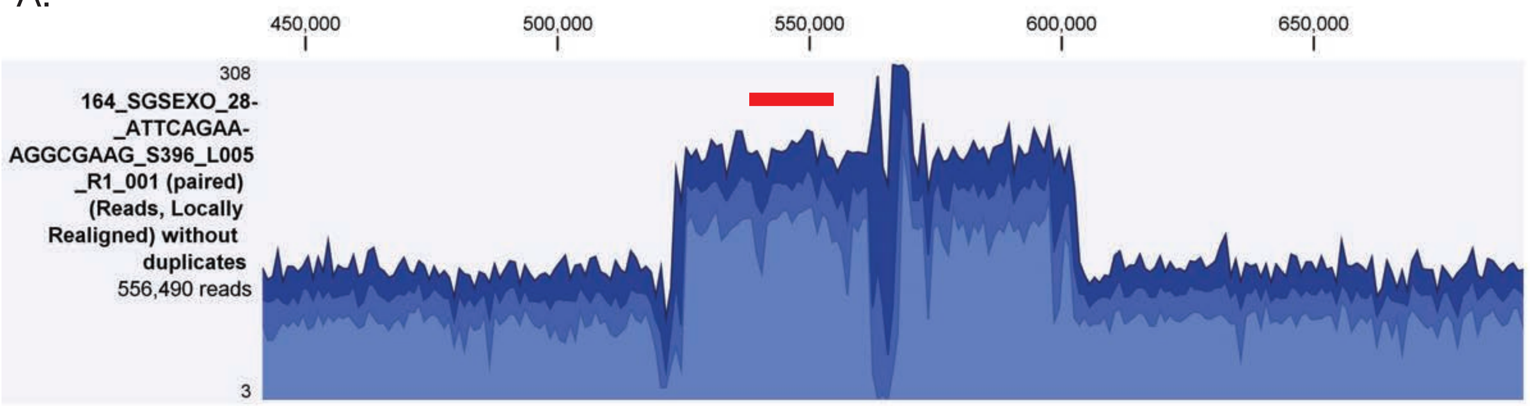

Sample_Barcode	Chromosome	Cluster_start	Cluster_end	Cluster_Length	Cluster_Size_Complexes	Mutation_Density_per_kb	CG_cluster_classification >3mutations
164_SGSEXO_28	chr14	538414	555320	16907	10	0.59	coord with terminal CG

B.

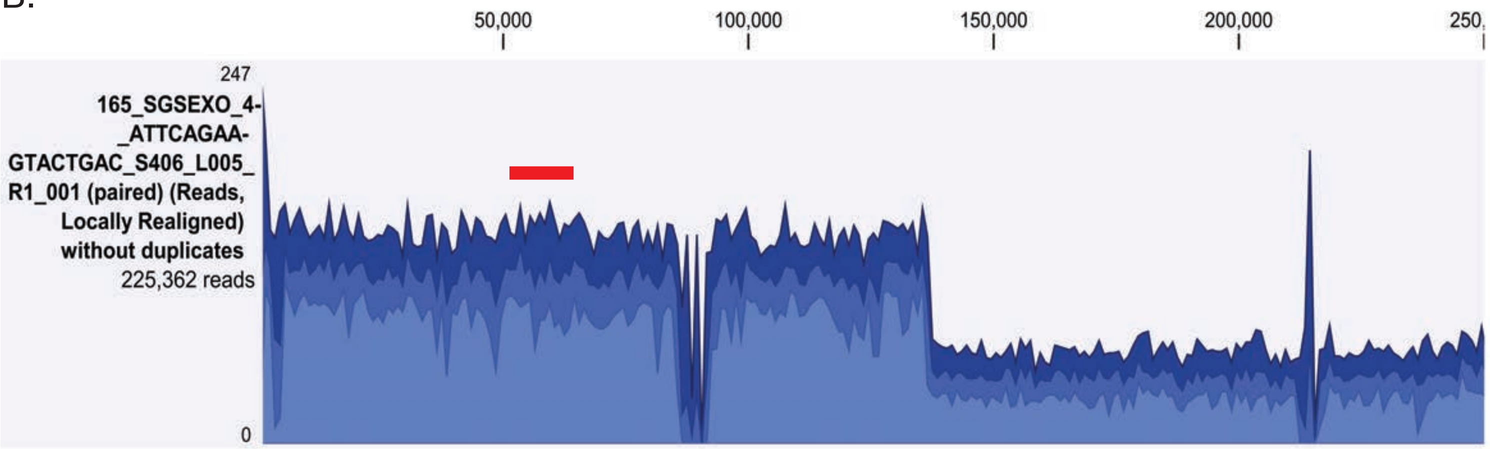

Sample_Barcode	Chromosome	Cluster_start	Cluster_end	Cluster_Length	Cluster_Size_Complexes	Mutation_Density_per_kb	CG_cluster_classification >3mutations
165_SGSEXO_4	chr8	53107	64198	11092	4	0.36	G- coord

C.

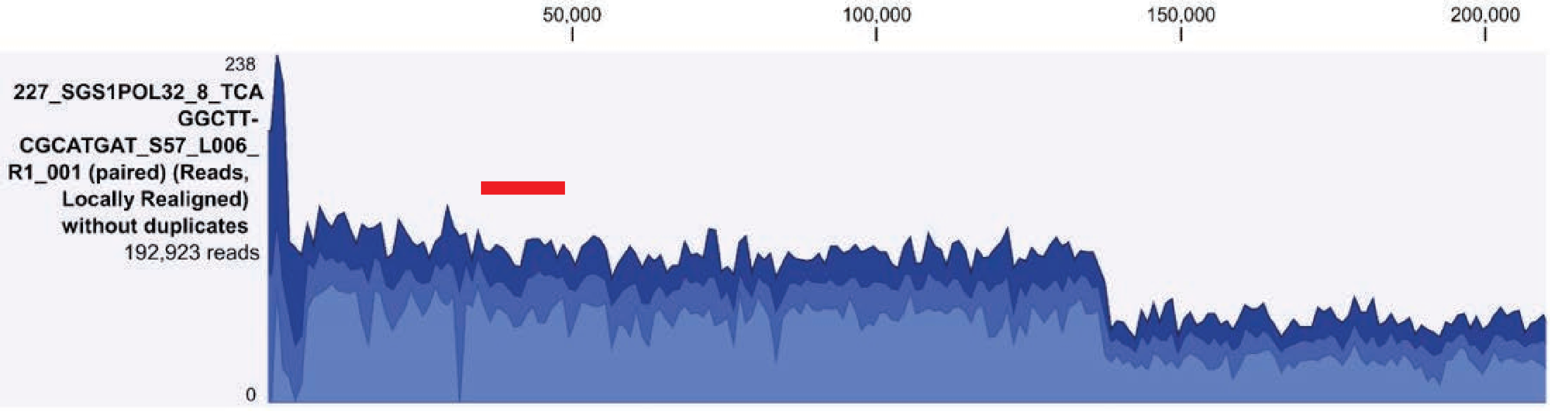

Sample_Barcode	Chromosome	Cluster_start	Cluster_end	Cluster_Length	Cluster_Size_Complexes	Mutation_Density_per_kb	CG_cluster_classification >3mutations
227_SGS1POL32_8	chr5	35773	48877	13105	15	1.14	CG single-switch
